# Cardiopulmonary function in special operations forces compared to conventional infantry soldiers

**DOI:** 10.1113/EP092993

**Published:** 2025-11-25

**Authors:** Rasmus Syberg Rasmussen, Jakob Solgaard Jensen, Iben Elmerdahl Rasmussen, Jacob Peter Hartmann, Clara Sofie Egeberg, Anna Agnes Lytzen, Klara Nielsen, Malte Lund Adamsen, Mads Fischer, Ronan M. G. Berg

**Affiliations:** ^1^ Centre for Physical Activity Research Copenhagen University Hospital – Rigshospitalet Copenhagen Denmark; ^2^ Department of Biomedical Sciences, Faculty of Health and Medical Sciences University of Copenhagen Copenhagen Denmark; ^3^ Department of Nutrition, Exercise and Sports University of Copenhagen Copenhagen Denmark; ^4^ Fertility Department 4071 Copenhagen University Hospital – Rigshospitalet Copenhagen Denmark; ^5^ Department of Cardiology University of Copenhagen, Bispebjerg Hospital Copenhagen Denmark; ^6^ Department of Clinical Physiology and Nuclear Medicine Copenhagen University Hospital – Rigshospitalet Copenhagen Denmark; ^7^ Department of Clinical Medicine, Faculty of Health and Medical Sciences University of Copenhagen Copenhagen Denmark; ^8^ Neurovascular Research Laboratory, Faculty of Life Sciences and Education University of South Wales Pontypridd UK

**Keywords:** echocardiography, lung function, maximal oxygen uptake, military medicine, training adaptations

## Abstract

Fitness is essential to military personnel in general, especially in the special operations forces (SOF), where the demanding tasks require a high level of physical fitness and mental robustness. However, little research has been done on SOF to characterise the putative underlying cardiopulmonary adaptations that distinguish them from conventional infantry soldiers (INF). This study aims to evaluate the cardiopulmonary function in SOF compared INF. The study assessed cardiac function and dimension using transthoracic echocardiography obtained at rest in eight soldiers from a SOF unit and in eight INF. ${\dot{V}}_{O_{2}\max}$ was measured by direct calorimetry (secondary outcome) at the same time blood samples were collected to measure lactate levels. Lung function was assessed by spirometry, while the haemoglobin‐corrected pulmonary diffusing capacity for carbon monoxide (*D*_L,COc_) was examined by the single‐breath technique. SOF had higher stroke volume (mean difference = 21 mL, *P *< 0.001) and left ventricular ejection fraction (mean difference = 7%, *P *= 0.026) than INF. Furthermore, SOF had higher global constructive myocardial work and global work index compared to INF. ${\dot{V}}_{O_{2}\max}$ as percentage of predicted according to age, weight and sex was higher in SOF, and they also had lower lactate levels during the ${\dot{V}}_{O_{2}\max}$ test than INF (*P *= 0.029). None of the measured lung function metrics differed between groups. In conclusion, when compared to conventional infantry soldiers, SOF soldiers had marked cardiac adaptations with evidence of eccentric LV remodelling. It remains to be determined if this reflects different training regimes or selection.

## Abstract (highlights)

- **What is the central question of this study?**
  Do special operations forces exhibit superior cardiopulmonary function compared to conventional infantry soldiers?
- **What is the the main finding and its importance?**
  Special operation forces exhibited evidence of eccentric left ventricular remodelling with a concomitant relatively high whole‐body maximal oxygen uptake. It remains to be determined whether this reflects their specific training regime or selection.

## 1 INTRODUCTION

Special operations forces (SOF) are trained to achieve exceptional strength and endurance, enabling them to execute demanding tasks such as carrying heavy loads, conducting hostage rescues, and responding to threats with minimal preparation time (Angeltveit et al., 2016; Maupin et al., 2018). Research indicates that the whole body maximal oxygen uptake (${\dot{V}}_{O_{2}\max}$) and strength levels of SOF units are comparable to that of elite athletes (Angeltveit et al., 2016; Maupin et al., 2018). Due to the distinct physical demands, SOF units likely have different physical fitness levels compared to conventional infantry soldiers, with SOF units focused on rapid deployments and infantry on monitoring and patrolling (Heinrich et al., 2012; Simpson et al., 2017; WesBrock et al., 2016).

${\dot{V}}_{O_{2}\max}$ has been compared between SOF units and infantry soldiers in previous studies, showing higher values in the former (Angeltveit et al., 2016; Figueir et al., 2022; Heinrich et al., 2012; Maupin et al., 2018 2018). However, the underlying cause of higher ${\dot{V}}_{O_{2}\max}$ in SOF remains to be determined, and may in principle reflect differences from conventional infantry soldiers in any step of the oxygen transport cascade (Wagner, 2023). However, a previous study of cardiac function found that SOF unit soldiers exhibit eccentric cardiac structural adaptations as observed in endurance athletes (Dinis et al., 2018), while many other studies indicate that endurance athletes exhibit relatively high standardised lung function metrics, including forced expiratory volume in one second (FEV_1_), forced vital capacity (FVC), total lung capacity (TLC), and pulmonary diffusing capacity for carbon monoxide (*D*_L,CO_) compared to non‐athletes (Peters et al., 2023; Thomsen et al., 2025).

Given the exceptional physical demands placed on SOF units, we sought to investigate whether there are differences in lung and cardiac function between SOF units and well‐trained infantry soldiers assessed in their routine non‐operational environment. We hypothesised that active SOF soldiers have structural and functional cardiac adaptations associated with intensive physical training and higher ${\dot{V}}_{O_{2}\max}$ compared to conventional infantry soldiers.

## 2 METHODS

### 2.1 Ethical approval

The study protocol was approved by the Scientific Ethical Committee of the Capital Region of Denmark (H‐22045997). The study conformed to the standards set by the *Declaration of Helsinki*, except for registration in a database. All participants gave oral and written informed consent prior to participation.

### 2.2 Participants

Eight SOF soldiers, and eight conventional infantry soldiers, both corresponding to a typical size of a squad, participated in the study. All participants were recruited at their place of service, and their commanding officer approved their participation. The SOF soldiers were recruited first due to limited availability. Infantry soldiers of comparable age were requested, but the request could not be fulfilled. In this study, SOF was defined as personnel operating in an established SOF unit who has completed all special training required to operate in the SOF unit, meaning they have at least 1 year of experience with active duty. A conventional infantry soldier was defined as a professional soldier who has completed training at the constable level and has at least 1 year of active duty in the military.

### 2.3 Design

Upon arrival at the study site, the participants were asked to fill out the international physical activity questionnaire (IPAQ) (long form). All participants underwent lung function measurements, a body dual energy X‐ray absorptiometry (DXA) scan, resting echocardiography, upper and lower body strength tests, and a ${\dot{V}}_{O_{2}\max}$ exercise test. All examinations were done in the same order for all participants, starting with lung function measurements. The participants fasted overnight but were allowed to eat and drink after the lung function measurements, DXA‐scan and echocardiography were completed.

### 2.4 Cardiopulmonary exercise test

Oxygen uptake was measured using a graded exercise test on a cycle ergometer (LC4, Monark Exercise AB, Vansbro, Sweden). The test started with 5 min warm up with a load (W) corresponding to twice the lean mass (kg). Warm‐up was immediately followed by a 25 W increase every minute until the participant reached volitional exhaustion. During the test, subjects were instructed to pedal at a self‐selected cadence greater than 60 rpm. Ventilation and expired gases were measured during the test using a Quark gas analyser (COSMED Quark cPET System, COSMED Srl, Rome, Italy), and heart rate was measured in beats per minute with a smartLAB device (Dossenheim, Germany) chest band, compatible with the COSMED system were assessed simultaneously. The COSMED system was calibrated prior to testing, including gas and flow calibration. The ${\dot{V}}_{O_{2}\max}$ test was considered valid when 2 out of 3 criteria were met: (1) respiratory exchange ratio (RER) of >1.1, (2) BORG scale (6–20) >17, and (3) plateau in oxygen uptake. Peak minute ventilation (${\dot{V}}_{\mathrm{Epeak}}$, L/min) and peak oxygen uptake (${\dot{V}}_{O_{2}\max}$, L/min) were defined as a mean of the highest 30 s during the exercise test. Peak power output (*W*_Lpeak_) was defined at the highest workload reached and completed during the exercise test. Peak respiratory exchange ratio (RER_peak_) and peak oxygen pulse (${\dot{V}}_{O_{2}}$/heart rate) were defined as the highest values obtained during the entire exercise test. ${\dot{V}}_{O_{2}\max}$ as percentage of predicted according to FRIEND prediction set based on age, weight, and sex was also calculated (Myers et al., 2017).

### 2.5 Lactate response to exercise

Blood samples were collected in a resting state on the cycle ergometer at time point 0, after 5 min of exercise, and then every minute until the end of the ${\dot{V}}_{O_{2}\max}$ test. After exhaustion was reached, participants were seated in a chair. Two final blood samples were collected 2 and 5 min after exhaustion, respectively. All blood samples were analysed immediately after collection (ABL825, Radiometer, Copenhagen, Denmark). The lactate response was expressed as area under the curve to reflect the integrated concentration–time profile across the incremental test, and thus the overall balance between lactate production and clearance while accounting for individual differences in test duration.

### 2.6 Transthoracic echocardiography

Transthoracic echocardiography (TTE) was performed using a GE Vivid E95 ultrasound machine with a 2.5‐MHz transducer (GE Healthcare, Chicago, IL, USA) following guidelines from the American Society of Echocardiography and the European Association of Cardiovascular Imaging (Lang et al., 2015). Measurements were obtained by experienced echocardiographers (J.S.J., M.F.) with participants positioned supine and in the left lateral position in a darkened room at 23°C. The frame rate was set to at least 57 frames per second to optimise spatial resolution. Analyses were performed using Echopac v206 and automated function imaging (AFI) 3.0 (GE Healthcare, Chicago, IL, USA). A minimum of three cardiac cycles were analysed for each measure and the mean was reported. Body surface area (BSA) was calculated using the DuBois formula (DuBois & DuBois, 1915). Relative wall‐thickness was assessed from the parasternal long axis view and calculated by the formula (2 × posterior wall‐thickness (cm))/Left ventricular internal dimension in diastole (cm).

Left ventricular (LV) and atrial volumes were calculated using Simpson's biplane method from two‐ and four‐chamber views. LV stroke volume/index, LV ejection fraction (LVEF), and cardiac output/index were derived from biplane LV end‐systolic and end‐diastolic volumes, combined with the respective heart rates. LV mass was assessed using the area–length method with traced epicardium and endocardium at the papillary muscle level in the parasternal short‐axis view. Analysis of the left ventricle, right ventricle and atrial segments was performed semi‐automatically using AFI and tracking speckles within the region of interest through cardiac cycles by tracing endocardial borders in the four‐chamber, two‐chamber and three‐chamber views across 17 segments. The mean resting blood pressure measurement of three systolic and diastolic blood pressures was used to assess myocardial work along with the strain analyses in accordance with acquiring measures of non‐invasive pressure strain‐loops (Russell et al., 2012). Right ventricular function was assessed by tricuspid annular plane systolic excursion (TAPSE) by M‐mode and AFI.

Eccentric structural assessment included LV mass, relative wall thickness and mean LV internal dimension. Apart from standard metrics of cardiac function, such as stroke volume and cardiac output, and LVEF, global longitudinal strain (GLS) was used to evaluate LV systolic function, and TAPSE to assess right ventricular systolic function. Regarding myocardial work, LV global constructive work (GCW) was used to assess myocardial work during the ejection phase and negative strain in the isovolumic relaxation phase of the cardiac cycle, while the LV myocardial global work index (GWI) was used to assess both constructive and wasted work, encompassing cardiac work during ejection and, to some extent, the isovolumetric phases of the cardiac cycle, depending on both myocardial contractility and left ventricular afterload. Global wasted work (GWW) was used to quantify the myocardial work that does not contribute to ejection, mainly representing energy expended during myocardial lengthening in systole and shortening during isovolumic relaxation.

### 2.7 Lung function

Standardised lung function testing was performed at a dedicated facility at Centre for Physical Activity Research (CFAS), and in accordance with consensus guidelines (Graham et al., 2017; Stanojevic et al., 2022). Testing involved dynamic spirometry, body plethysmography, and single‐breath carbon monoxide uptake, for determining FEV_1_, FVC, TLC, residual volume (RV), and haemoglobin‐corrected *D*_L,CO_ (*D*_L,COc_). The prediction set used for these lung function metrics was Standard EU GLI.

### 2.8 DXA scan

A total DXA (Lunar Prodigy, GE Healthcare, Madison, WI, USA). Encore software version 14, 10, 0022) was used to determine total fat mass, fat mass percentage and lean body mass. All subjects were asked to void prior to the scan.

### 2.9 Strength test

Hand grip strength was assessed after two initial tests were conducted to adapt to the equipment. Afterwards, the test began, with each participant having three attempts. The highest force recorded for each hand was registered. Upper and lower body strength were assessed by one‐repetition maximum (1RM) tests defined as maximum amount of weight that can be lifted once with proper form through full range of motion = 1RM. The subjects were tested in chest press and leg press machines (Technogym Runrace, Gambettola, Italy).

For chest press and leg press 1RM tests, subjects performed progressively fewer and heavier loads before attempts of a 1RM. Between each attempt 2–3 min of rest was required. If the heaviest possible load of the machine was reached, a maximum number of repetitions was conducted on the heaviest load and strength was estimated according to percentage of 1RM maximum (Hoffman, 2014). If more than 10 repetitions were completed on the maximum weight of the machine, 10 repetitions maximum was estimated from the test due to the limitations related to estimating 1RM from >10 repetitions (Hoffman, 2014).

### 2.10 Statistical analyses

Unless otherwise stated, data are presented as means (SD). Parametric tests were used for between‐group comparisons after ensuring a normal distribution of data by visual histogram inspection. The statistical analysis of TTE was conducted in R (R studio 2024.12.1; R for windows 4.4.2), while the statistical analyses of all remaining parameters were conducted in Graphpad Prism (Version 10.4.1 (532); GraphPad Software, Boston, MA, USA). For all data, a two‐sided *P *< 0.05 was considered statistically significant.

## 3 RESULTS

### 3.1 Participant characteristics

Eight SOF soldiers and eight conventional infantry soldiers were included. Participant characteristics are shown in Table 1. Comparing anthropometric and physical parameters, SOF soldiers were older, had higher lean body mass, and lower body fat percentage compared to conventional infantry soldiers. SOF soldiers had higher resting systolic, diastolic and mean arterial blood pressures than conventional infantry soldiers, as well as a lower resting heart rate. All participants in the SOF group had a high level of physical activity, assessed via IPAQ, in their daily life. In the group of conventional infantry soldiers, seven had a high level of physical activity in their daily life, and one had a moderate physical activity level.

**TABLE 1:** Participant characteristics.

	SOF (*n* = 8)	INF (*n* = 8)	Between‐group difference	*P*
Age (years)	34 (5.6)	26 (1.7)	8.0	0.004
Height (cm)	183.0 (3.5)	181.9 (6.9)	1.1	0.695
Weight (kg)	85.5 (4.0)	87.5 (8.4)	2.0	0.560
BMI (kg/m^2^)	25.6 (1.6)	26.5 (1.9)	0.9	0.533
Fat mass (%)	13.4 (3.8)	19.7 (4.5)	6.26	0.009
Lean body mass (kg)	70.8 (2.7)	66.9 (4.7)	3.9	0.068
Systolic BP (mmHg)	136.8 (13.6)	119.5 (8.4)	17.3	0.008
Diastolic BP (mmHg)	77.8 (6.7)	70.4 (4.1)	7.4	0.017
MAP (mmHg)	97.5 (7.9)	86.8 (4.4)	10.7	0.007
Resting heart rate (bpm)	52 (7)	54 (9)	1	0.758

Participant characteristics reported as mean (SD). Abbreviations: BMI: body mass index; BP: blood pressure; INF: conventional infantry soldiers; MAP: mean arterial blood pressure; SOF: special operations forces.

### 3.2 Cardiac function

**FIGURE 1:**
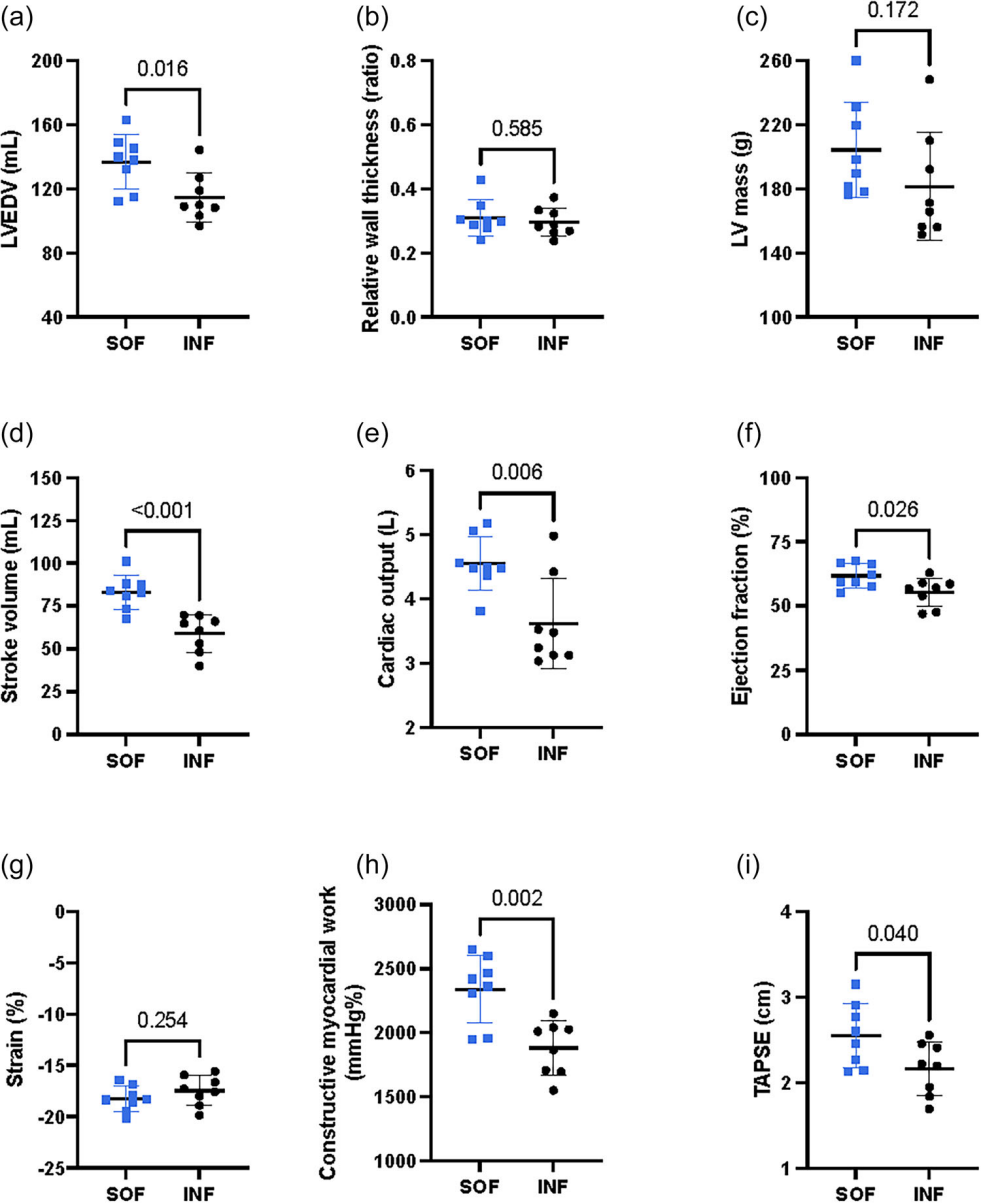
Echocardiography during rest in special operations forces (SOF) vs. conventional infantry soldiers (INF). Individual data points are shown as dots (blue represents SOF, *n* = 8; black represents INF, *n* = 8). Horizontal lines indicate means with error bars representing SD. LV, left ventricular; LVEDV, left ventricular end‐diastolic volume. TAPSE, tricuspid annular plane systolic excursion.

The SOF group exhibited a higher stroke volume, cardiac output and LVEF than conventional infantry soldiers (Figure 1a–c). The SOF group had a higher left ventricular end‐diastolic volume (Figure 1d) and left ventricular internal dimension (5.6 (0.34) cm vs. 5.3 (0.29) cm; indexed 2.7 (0.16) cm/m^2^ vs. 2.5 (0.13) cm/m^2^, *P* = 0.032), but similar LV mass and relative wall thickness (Figure 1e–f). With no difference in GLS, GCW was higher in the SOF group compared to the conventional infantry soldiers (Figure 1i), with corresponding differences for GWI (2000 (260) mmHg % vs. 1700 (210) mmHg %, *P* = 0.020) and GWW (120 (45) mmHg % vs. 80 (21) mmHg %, *P* = 0.046).

### 3.3 ${\dot{V}}_{O_{2}\max}$, lactate threshold and strength

All ${\dot{V}}_{O_{2}\max}$ tests fulfilled the a priori defined validation criteria. While neither absolute nor relative ${\dot{V}}_{O_{2}\max}$ differed between groups, ${\dot{V}}_{O_{2}\max}$ %pred was higher in the SOF group (Table 2). The cardiopulmonary exercise test to exhaustion lasted on average 13.9 min, ranging from 12.0 min to 15.4 min, in SOF soldiers and on average 12.5 min, ranging from 10.7 min to 14.1 min, in conventional infantry soldiers (*P* = 0.034).

**TABLE 2:** Cardiopulmonary exercise test results.

	SOF (*n* = 8)	INF (*n* = 8)	Between‐group difference	*P*
Absolute ${\dot{V}}_{O_{2}\max}$ (mL/min)	4169 (324)	3838 (749)	331	0.272
Relative ${\dot{V}}_{O_{2}\max}$ (mL/min/kg)	48.9 (4.9)	43.9 (8.0)	5	0.155
${\dot{V}}_{O_{2}\max}$ %pred (%)	114 (9.7)	96 (17)	18	0.002
${\dot{V}}_{\mathrm{Epeak}}$ (L/min)	186 (19)	167 (31)	19	0.151
${\dot{V}}_{\mathrm{Epeak}}$/MVV (%)	84 (11)	74 (8)	10	0.058
HR_peak_ (beats/min)	187 (9.7)	193 (9.2)	6	0.272
*W*_L,peak_ (W)	376 (21)	314 (50)	62	0.006
Peak ${\dot{V}}_{O_{2}}$/HR (mL/beat)	22.8 (2)	23.2 (8)	0.4	0.906
RER_peak_	1.27 (0.04)	1.20 (0.09)	0.07	0.081

Cardiopulmonary exercise test results in special operations forces (SOF) vs. conventional infantry soldiers (INF) reported as mean (SD). Abbreviations: HR_peak_, peak heart rate; MVV, maximal voluntary ventilation; ${\dot{V}}_{O_{2}}$/HR, oxygen pulse (whole‐body oxygen uptake per heartbeat); RER_peak_, peak respiratory exchange ratio; ${\dot{V}}_{\mathrm{Epeak}}$, peak minute ventilation. ${\dot{V}}_{O_{2}\max}$, whole‐body maximal oxygen uptake; ${\dot{V}}_{O_{2}\max}$ %pred, ${\dot{V}}_{O_{2}\max}$ as percentage of predicted according to sex, age, and weight; *W*_L,peak_: peak power output.

**FIGURE 2:**
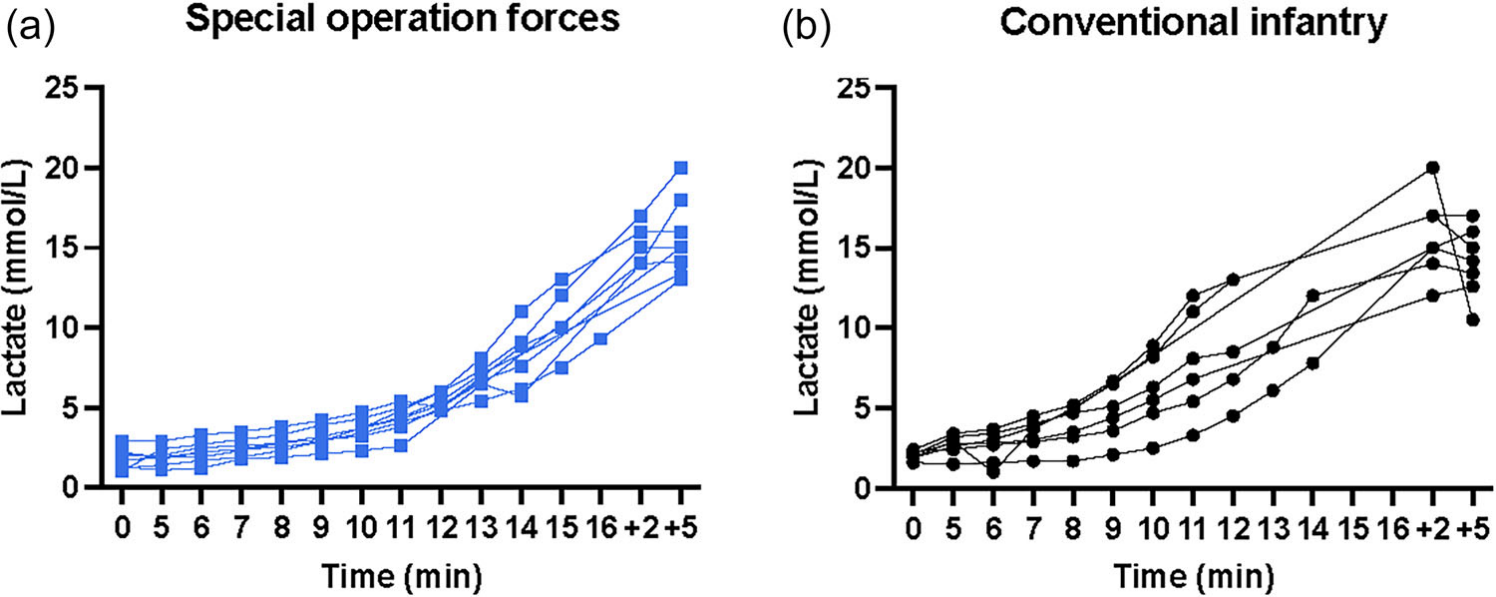
Lactate levels during an incremental cardiopulmonary exercise test in (a) special operations forces and (b) conventional infantry soldiers. The data points +2 and +5 refers to the mean lactate levels at 2 and 5 min after exhaustion, respectively.

During the incremental cardiopulmonary exercise test, the SOF group exhibited a lower lactate response compared with the conventional infantry group (85.5 (9.9) mmol/L min vs. 115.6 (25.5) mmol/L min, *P* = 0.029) (Figure 2). After exclusion of one individual in the conventional infantry group whose baseline serum lactate was 9.0 mmol/L (approximately five‐fold above normal range and therefore likely due to equipment error), the mean lactate concentrations at 2 and 5 min after exhaustion were 15.2 (1.3) mmol/L and 15.5 (2.4) mmol/L in the SOF group and 15.7 (2.4) mmol/L and 14.2 (2.0) mmol/L in the conventional infantry group, respectively (Figure 2).

No between‐group differences in muscular strength were observed (Table 3).

**TABLE 3:** Strength test results.

	SOF (*n* = 8)	INF (*n* = 8)	Between‐group difference	*P*
Grip strength left (kg)	61.4 (8.0)	54.1 (7.5)	7.3	0.081
Grip strength right (kg)	59.9 (6.2)	57.9 (9.0)	2	0.611
Chest press (kg)	130.9 (10.6)	120.2 (17.6)	10.7	0.161
Leg press (kg)	309.5 (46.9)	260.0 (67.1)	49.5	0.109

Strength assessment of special operations forces (SOF) vs. conventional infantry soldiers (INF) reported as mean (SD).

### 3.4 Lung function

The lung function results are provided in Table 4. There were no differences in any lung function metrics between groups.

**TABLE 4:** Lung function test results.

	SOF (*n* = 8)	Conventional infantry (*n* = 8)	Between‐group difference	*P*
FEV_1_ %pred (%)	119.6 (8.9)	114.9 (13.0)	4.7	0.391
FVC %pred (%)	120.1 (8.3)	116.9 (9.6)	3.2	0.481
TLC %pred (%)	107.0 (8.2)	100.9 (7.9)	6.1	0.152
RV %pred (%)	103.4 (24.6)	81.5 (16.8)	21.9	0.057
*D*_L,COc_ %pred (%)	109.2 (18.6)	96.7 (5.6)	12.5	0.091

Lung function in special operations forces (SOF) vs. conventional infantry soldiers (INF) reported as mean (SD). Prediction set: standard EU GLI. Abbreviations: *D*_L,COc_, haemoglobin‐corrected pulmonary diffusing capacity for carbon monoxide; FEV_1_, forced expiratory volume in 1 s; FVC, forced vital capacity; pred, predicted; RV, residual volume; TLC, total lung capacity.

## 4 DISCUSSION

In the present study, operational SOF soldiers, assessed under a routine non‐operational environment, exhibited distinct cardiac structural and functional adaptations compared to conventional soldiers. SOF soldiers had higher stroke volume and LVEF. GLS was similar between the groups, but the SOF group displayed overall higher myocardial work.

Contrary to our expectations based on previous literature (Angeltveit et al., 2016; Figueir et al., 2022; Heinrich et al., 2012; Maupin et al., 2018; Simpson et al., 2017), the between‐group differences in both absolute and relative ${\dot{V}}_{O_{2}\max}$ were not statistically significant. However, the SOF group exhibited a higher ${\dot{V}}_{O_{2}\max}$ %pred, i.e., when accounting for age and body weight (but not sex, all participants being male), compared with the conventional infantry group. A likely confounder at play here is probably age, as also highlighted in a previous review on fitness profiles in elite tactical units, including both SOF and SWAT teams (Maupin et al., 2018). In this review, the mean relative ${\dot{V}}_{O_{2}\max}$ across six studies on a total of 211 individuals varied from 45 to 60 mL/min/kg, with age being highlighted as a confounder in this context. Somewhat lower ${\dot{V}}_{O_{2}\max}$ values have been reported in other military personnel, including conventional infantry soldiers, which are generally between 41 and 50 mg/min/kg (Figueir et al., 2022; Heinrich et al., 2012), but to the best of our knowledge, no direct comparisons to SOF have previously been provided. Apart from the confounding effect of age, the lack of a statistically significant between‐group difference, particularly in relative ${\dot{V}}_{O_{2}\max}$, may also reflect a type II error (Berg et al., 2024), potentially attributable to the relatively small sample size, as well as a high variability of ${\dot{V}}_{O_{2}\max}$ values within the comparator group. The latter may at least to some extent reflect greater inconsistency in physical training regimens among conventional infantry soldiers.

The lower lactate levels in the SOF group during the incremental cardiopulmonary exercise test was surprising, especially when considering the ∼1.5 min longer duration of the tests in the SOF soldiers (Figure 2). This may reflect a greater metabolic capacity in the working skeletal muscle, i.e. due to enzymatic composition, mitochondrial density and function, and/or fibre type composition, leading to a tighter coupling between cytosolic ATP turnover and mitochondrial oxidative phosphorylation in SOF soldiers. Such adaptations have previously been related to combined high‐volume training and high intensity training (Laursen, 2010). Alternatively, it may reflect superior clearance of lactate in the SOF group, which is a well‐established adaptation to both regular high‐intensity interval training and moderate‐intensity continuous training (Xie et al., 2024), and which is known to be a major factor when exercising while carrying a heavy backpack (Simpson et al., 2017). However, to conclude to what extent the lower lactate levels observed in the SOF group when compared to conventional infantry soldiers reflect reduced production or enhanced clearance would ideally require both morphological and enzymatic analyses of muscle biopsies, as well as detailed tracer kinetic analyses (Messonnier et al., 2013). In any event, the lower lactate at matched workloads in SOF is compatible with a higher lactate threshold and possibly a higher critical power – both established determinants of sustaining higher absolute work rates before exercise intolerance (Poole et al., 2016) – and may therefore confer advantages in SOF‐specific tasks, requiring prolonged load carriage, repeated high‐intensity efforts, and sustained movement under load.

The SOF group displayed higher stroke volume and LVEF compared to infantry soldiers, despite similar GLS, indicating other involving factors than myocardial contractility. Although the SOF group was older, normative echocardiographic data indicate that ageing from the third to the fourth decade is associated with slightly smaller LV volumes and lower stroke volume with no change in LVEF (Bernard et al., 2017; Patel et al., 2021; Peverill, 2021); thus, the higher stroke volume we observed in SOF is unlikely to be age‐driven. A more plausible explanation is therefore structural adaptations (e.g. higher LV end‐diastolic volume and internal dimensions) to intensive training, which has previously been reported to trigger eccentric LV remodelling in trained military personnel (Arbab‐Zadeh et al., 2014; Carrick‐Ranson et al., 2012; Fischer et al., 2025; Junianto et al., 2023).

The higher myocardial work in SOF soldiers at rest, as documented by higher GCW, GWI and GWW, indicates involvement of both the LV ejection phase and the isovolumetric phases of the cardiac cycle. To some extent, this may be influenced by the higher diastolic blood pressure, and thus higher LV afterload, observed in the SOF group. Similar findings have been provided in previous studies, in which the higher intensity of physical training introduced as part of SOF training leads to higher LV volumes, GLS, GCW and GWI and lower GWW than observed in regular troops and non‐military subjects. (Junianto et al., 2023). The typical training regime in SOF includes carrying of heavy items across longer distances, parachuting, swimming and overall hybrid training (Angeltveit et al., 2016; Maupin et al., 2018), which is in contrast to the typical training for infantry soldiers consisting of high amounts of low/moderate‐intensity training combined with strength training (Kyröläinen et al., 2018). This might explain the cardiac adaptations related to the SOF soldiers. As such, their training regimens reflect two different tasks when deployed, where the SOF soldiers are expected to be deployed within a short period of time and complete more highly intense varying tasks and the conventional infantry soldiers are expected to complete specific tasks like monitoring of an area and patrolling (Heinrich et al., 2012; Simpson et al., 2017; WesBrock et al., 2016). Looking into their resilience, mental and physical health studies suggest that SOF units are generally healthier than other military personnel, probably due the higher demands at the selection process (Turnley et al., 2018).

The soldiers, especially the SOF soldiers, were stronger than anticipated (Maupin et al., 2018), resulting in some soldiers doing >10 repetitions at the highest possible weight setting on the equipment. 1RM was thus estimated for these participants. However, as the 1RM estimates were associated with large uncertainties when calculated with >10 repetitions, 1RM values were estimated with no more than 10 repetitions – also for those who performed >10 repetitions. Hence, for some of the participants, the 1RM was underestimated, potentially explaining why no differences were detected between groups.

Lung function was similar between SOF and conventional infantry soldiers across all standard dynamic and static lung volumes as well as diffusing capacity measures reported here. Accordingly, a previous study found normal *D*_L,CO_ in SOF divers (den Ouden et al., 2022). However, it is notable that most of the participants in the present study had FEV_1_, FVC, TLC, and *D*_L,COc_ values above 100% of predicted according to age, sex and height. Similarly highly standardised lung function metrics are known to be present in healthy athletes, particularly in swimmers and cross‐country skiers (Thomsen et al., 2025). As such, neither of the studied groups here appears to differ markedly from non‐military athletes (den Ouden et al., 2022).

It is important to consider that the SOF solders were approximately 8 years older than the comparator group. Generally the peak aerobic performance of competitive athletes is observed at the age of 20–30 years (Tanaka & Toussaint, 2023), and ${\dot{V}}_{O_{2}\max}$ decreases gradually with advancing age, by approximately 10% per decade after the age of 25 years, even in trained individuals (Wilson & Tanaka, 2000). Thus, one could assume that SOF soldiers would exhibit significantly higher absolute and relative ${\dot{V}}_{O_{2}\max}$ values if they had been compared to age‐matched conventional infantry soldiers, which is also supported by the higher ${\dot{V}}_{O_{2}\max}$ %pred in the SOF group. While the present findings are consistent with the SOF training regime leading to both eccentric cardiac remodelling and concomitant changes in aerobic capacity, the design of this study does not permit us to conclusively determine whether this can be directly attributed to the training regime or whether they reflect selection, considering the extensive selection process required to qualify for SOF. Other limitations are that the age difference may also have confounded the observed between‐group differences (or lack thereof) for the various other measured parameters; that the echocardiographic assessments of cardiac function were done at rest only – exercise measurements would have been favourable here; that the strength testing equipment was insufficient for the purposes of this study with resultant underestimated 1RM values; and perhaps most importantly, the small sample size for both groups, which increases the risk of type II error (Berg et al., 2024). Another important limitation of this study is that only men were included. A male predominance in biomedical research has been widely recognised, with experiments on male animals and participants often exceeding those on females, despite guidelines promoting sex balance in both preclinical and clinical work (Vanden Noven et al., 2023). Because sex influences cellular function and the regulation of physiological systems, results obtained exclusively in men cannot automatically be extrapolated to women (Sheel, 2016). The absence of female participants therefore restricts the generalisability of our findings. This reflects a practical constraint, as there are still very few women serving in SOF units, even though numbers are increasing (U.S. Government Accountability Office, 2022).

In conclusion, SOF soldiers exhibited marked cardiac adaptations, with higher SV at rest and signs of eccentric LV remodelling, compared to professional soldiers from the conventional infantry. Both groups exhibited high standardised lung function metrics, as is typical for athletes. It remains to be determined whether the observed cardiac adaptations observed in SOF soldiers reflect their specific training regime or selection.

## AUTHOR CONTRIBUTIONS

Rasmus Syberg Rasmussen: Conception, design, data collection, data analysis, data interpretation, figures, first draft, revisions. Jakob Solgaard Jensen: data collection, data analysis, data interpretation, first draft, revisions. Iben Rasmussen: design, data collection, revisions. Jacob Peter Hartmann: data collection, revisions. Clara Sofie Egeberg: data collection, revisions. Anna Agnes Lytzen: data collection, revisions. Klara Nielsen: Design, figures, revisions. Malte Adamsen: data collection, revisions. Mads Fischer: data collection, data interpretation, revisions. Ronan M. G. Berg: Conception, design, revisions, supervision. All authors have read and approved the final version of this manuscript and agree to be accountable for all aspects of the work in ensuring that questions related to the accuracy or integrity of any part of the work are appropriately investigated and resolved. All persons designated as authors qualify for authorship, and all those who qualify for authorship are listed.

## CONFLICT OF INTEREST

Prior to submission, the manuscript was reviewed and approved by the military units involved. No authors have any conflict of interests to declare.

## Data Availability

The data underlying this study are not publicly available due to the sensitive nature of the information and concerns regarding participant confidentiality.
